# Gut Microbiota Composition of Insectivorous Synanthropic and Fructivorous Zoo Bats: A Direct Metagenomic Comparison

**DOI:** 10.3390/ijms242417301

**Published:** 2023-12-09

**Authors:** Igor V. Popov, Ilia V. Popov, Anastasya A. Krikunova, Tatyana A. Lipilkina, Tatyana N. Derezina, Michael L. Chikindas, Koen Venema, Alexey M. Ermakov

**Affiliations:** 1 Faculty of Bioengineering and Veterinary Medicine and Center for Agrobiotechnology, Don State Technical University, 344000 Rostov-on-Don, Russia; ivpopov@donstu.ru (I.V.P.); akrikunova@donstu.ru (A.A.K.); tlipilkina@donstu.ru (T.A.L.); derezinasovet@mail.ru (T.N.D.); tchikind@sebs.rutgers.edu (M.L.C.); amermakov@yandex.ru (A.M.E.); 2 Division of Immunobiology and Biomedicine, Center of Genetics and Life Sciences, Sirius University of Science and Technology, 354340 Federal Territory Sirius, Russia; 3 Centre for Healthy Eating & Food Innovation (HEFI), Maastricht University Campus Venlo, 5928 SZ Venlo, The Netherlands; k.venema@maastrichtuniversity.nl; 4 Health Promoting Naturals Laboratory, School of Environmental and Biological Sciences, Rutgers State University, New Brunswick, NJ 08901, USA; 5 Department of General Hygiene, I.M. Sechenov First Moscow State Medical University, 119435 Moscow, Russia

**Keywords:** bat, gut microbiota, 16S rRNA, metagenome

## Abstract

Bats are natural reservoirs for many emerging viral diseases. That is why their virome is widely studied. But at the same time, studies of their bacterial gut microbiota are limited, creating a degree of uncertainty about the role of bats in global microbial ecology. In this study, we analyzed gut microbiota of insectivorous *Nyctalus noctula* and *Vespertilio murinus* from rehabilitation centers from Rostov-on-Don and Moscow, respectively, and fructivorous *Carollia perspicillata* from the Moscow Zoo based on V3–V4 16S rRNA metagenomic sequencing. We revealed that microbial diversity significantly differs between the insectivorous and fructivorous species studied, while the differences between *N. noctula* and *V. murinus* are less pronounced, which shows that bats’ gut microbiota is not strictly species-specific and depends more on diet type. In the gut microbiota of synanthropic bats, we observed bacteria that are important for public health and animal welfare such as *Bacteroides*, *Enterobacter*, *Clostridiaceae*, *Enterococcus*, *Ureaplasma*, *Faecalibacterium*, and *Helicobacter*, as well as some lactic acid bacteria such as *Pediococcus*, *Lactobacillus*, *Lactococcus*, and *Weisella*. All these bacteria, except for *Bacteroides* and *Weisella,* were significantly less abundant in *C. perspicillata*. This study provides a direct metagenomic comparison of synanthropic insectivorous and zoo fructivorous bats, suggesting future directions for studying these animals’ role in microbial ecology.

## 1. Introduction

Chiroptera is one of the most diverse orders of mammals, which includes more than 1400 species and represents approximately 20% of the world’s mammal population [1,2]. A relatively long lifespan, the ability to fly, unique immune responses, and their microbiome distinguish bats from other mammals [3,4,5].

In recent years, bats have received increasing attention from scientists as they are recognized as a potential natural reservoir for many emerging diseases. Nipah and Hendra viruses, Ebola-related viruses, SARS-CoV, MERS-CoV, and SARS-CoV-2 are some of the most notorious viruses that wreaked havoc on public health. Several studies show that these viruses have a bat origin, or some bat species could be at least an intermediate reservoir of transmissible infections caused by these viruses [6,7,8,9,10,11].

As most of the bat-derived emerging pathogens have a viral origin, the virome of bats is studied more than their bacterial or fungal microbiota [10]. Nevertheless, studies show that bats are hosts to fungi and bacteria, which are also critical for public health and animal welfare [12,13]. The wide diversity of species in the Chiroptera order makes it difficult to identify bat species-specific microbiomes. Considering that some studies show that the gut microbiota composition of bats also depends on their habitat location and diet type [14], this task becomes nearly impossible. However, the close relationship between the habitat of bats and their microbiota further demonstrates that these animals are an integral part of the global microbial ecology.

The strong dependence of the bat gut microbiota on the environment could be related to their ability to fly, which led to the overall shortening of their gastrointestinal tract (GIT) during their evolution. Some bat species lack a caeca [15,16] and an ascending and transverse colon [16], resulting in a shorter GIT compared to animals of the same size [17]. A short GIT and the absence of some of its parts result in relatively shorter food digestion time and, most importantly, in lower anaerobic volumes, which in turn provide unfavorable conditions for anaerobic bacteria and increase the proportion of transient environmental microbes [18]. All the above-mentioned characteristics, combined with the ability to fly long distances and a long lifespan, make bats unpredictable carriers of various microbes.

Our study aimed to characterize the gut microbiota composition of synanthropic insectivorous bat species *Nyctalus noctula* and *Vespertilio murinus*, for which samples were obtained from the bat rehabilitation centers of Rostov-on-Don and Moscow, respectively, and zoo fructivorous species *Carollia perspicillata* and make a direct statistical comparison of their features, such as richness, diversity, taxa abundance, and the abundance of the predicted functional pathways.

## 2. Results

### 2.1. Gut Microbiota Composition

**Figure 1:**
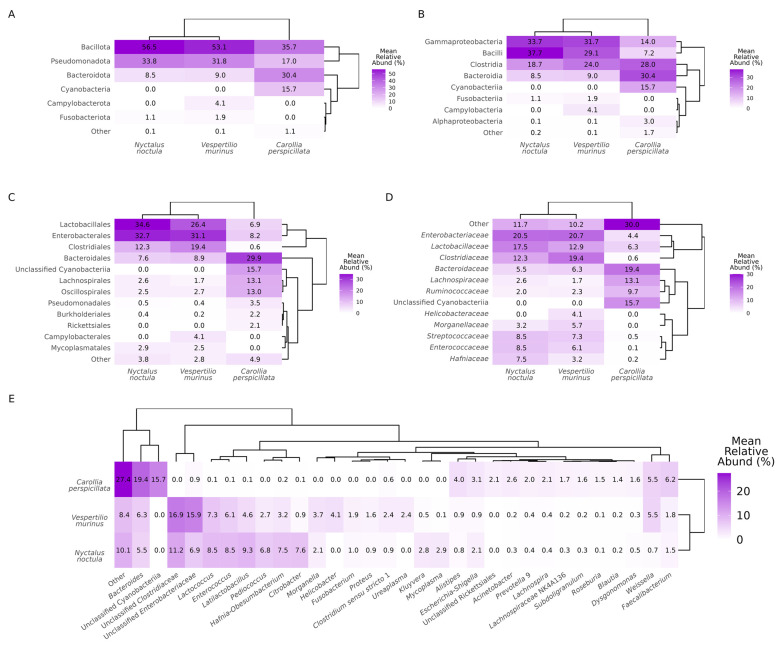
Heatmaps representing gut microbiota composition (as relative abundance (RA) of bacterial taxa) in samples from the *Nyctalus noctula*, *Vespertilio murinus*, and *Carollia perspicillata* species. RA at (**A**) phylum, (**B**) class, (**C**) order, (**D**) family, and (**E**) genus levels. Dendrograms represent clustering analysis based on the taxa RA similarities.

To investigate gut microbiota composition in *N. noctula*, *V. murinus*, and *C. perspicillata*, we sequenced amplicons of the V3–V4 regions of the 16S rRNA gene from metagenomic DNA isolated from fecal samples. Then, the classification of the sequences into amplicon sequence variants with further taxonomical identification on phylum, class, order, family, and genus levels and calculation of relative abundances (RAs) were performed. As a result, on the phylum level, we observed Bacillota, Pseudomonadota, and Bacterioidota in all three studied bat species. In samples from *C. perspicillata*, 15.7% of the observed sequences were identified as unclassified Cyanobacteria at all taxonomic levels. Additionally, on the phylum level, Fusobacteriota were observed in *N. noctula* and *V. murinus*, while Campilobacteriota was observed exclusively in *V. murinus*. Other phyla were observed in less than 1% of RA (Figure 1A). On the class level, Gammaproteobacteria, Bacilli, Clostridia, and Bacteroidia were observed in all three species studied, while Campylobacteria was identified only in *V. murinus*. Alphaproteobacteria was also detected in all three bat species studied, although its RA in insectivorous bats was less than 1%. Fusobacteria, as well as the phylum Fusobacteriota, were observed in *N. noctula* and *V. murinus* (Figure 1B). On the order level, the major abundant taxa that were observed in all studied bats were Lactobacillales, Enterobacterales, Clostridiales, Bacteroidales, Lachnospirales, Oscillospirales, Pseudomonadales, and Burkholderiales. Rickettsiales were observed only in *C. perspicillata* gut microbiota, Campylobacteriales in samples from *V. murinus*, and Mycoplasmatales only in studied insectivorous bats (Figure 1C). *Enterobacteriaceae*, *Lactobacillaceae*, *Clostridiaceae*, *Bacteroidaceae*, *Lachnopiraceae*, *Ruminococceae*, *Streptococcaceae*, and *Enterococcaceae* were among the most abundant families observed in all bat species studied, although the RA of some families varied several folds between species (Figure 1D). It is worth mentioning that the family *Helicobacteraceae*, as well as the genus *Helicobacter,* were observed exclusively in *V. murinus*. Genera with an RA greater than 1% in the gut microbiota of insectivorous bats were *Bacteroides*, unclassified *Clostridiaceae*, unclassified *Enterobacteriaceae*, *Lactococcus*, *Enterococcus*, *Latilactobacillus*, *Pediococcus*, *Hafnia-Obesumbacterium*, *Morganella*, *Fusobacterium*, and *Faecalibacterium*. Additionally, among genera with more than 1% RA, there were *Helicobacter*, *Proteus*, *Clostridium sensu stricto* 1, *Ureaplasma*, and *Weisella* in the gut microbiota of *V. murinus* and *Citrobacter*, *Kluyvera*, *Mycoplasma*, and *Escherichia-Shigella* in *N. noctula*. In samples from *C. perspicillata,* the following genera with an RA greater than 1% were present: *Bacteroides*, Alistipes, *Escherichia*-*Shigella*, *Acinetobacter*, *Prevotella* 9, *Lachnospira*, *Lachnospiraceae* NK4A136, *Subdoligranulum*, *Roseburia*, *Blautia*, *Dysgonomonas*, *Weisella*, and *Faecalibacterium* (Figure 1E).

**Figure 2:**
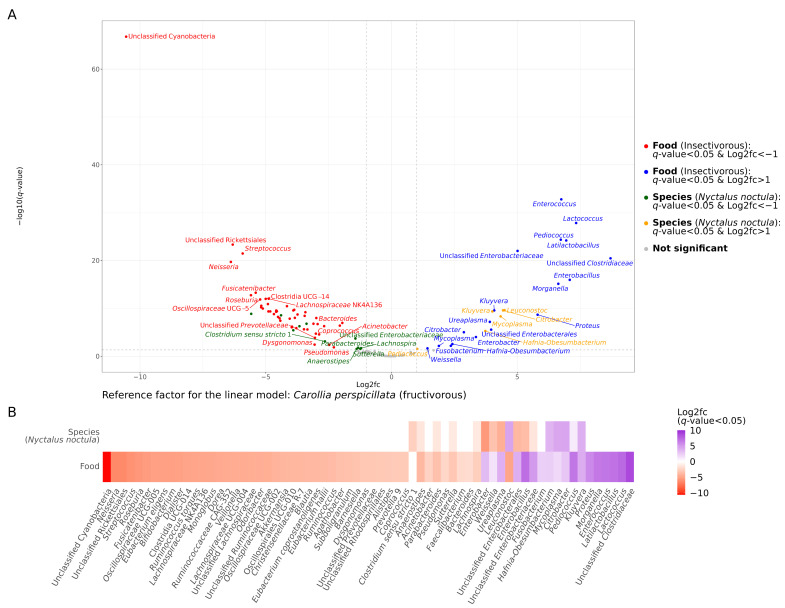
Results of the differential abundance analysis of bacterial genera identified in fecal samples of insectivorous (*Nyctalus noctula* and *Vespertilio murinus*) and fructivorous (*Carollia perspicillata*) bats. The fructivorous species *Carollia perspicillata* was used as a reference factor for the linear model analysis. (**A**) Volcano plots with genera, the relative abundance (RA) of which had significant associations with food type and studied bat species. Blue dots represent genera for which the RA was strongly associated with the insectivorous diet of bats. Red dots represent genera that were significantly less abundant or even absent in insectivorous bats compared to fructivorous ones. Yellow dots show genera that had a greater RA in *Nyctalus noctula* compared to *Carollia perspicillata* and *Vespertilio murinus*, while green dots represent genera, which were less abundant in *Nyctalus noctula* compared to *Carollia perspicillata* and *Vespertilio murinus*. (**B**) Heatmap with genera, the RA of which had significant associations with food type and studied bat species. In the top row, the purple cells in the heatmap represent genera for which the RA was significantly higher in *Nyctalus noctula* compared to *Carollia perspicillata* and *Vespertilio murinus*, while the orange cells show the opposite. In the second line, the purple cells represent genera with an RA significantly higher in the insectivorous bats studied compared to the fructivorous bats, and the orange cells show genera, which are significantly less abundant or absent in insectivorous bats compared to fructivorous bats.

To compare the gut microbiota composition of the studied bats, we performed a differential abundance analysis with the MaAsLin2 package [19] using linear models and fructivorous *C. perspicillata* as the reference factor. As a result, we found more pronounced differences in the RA of bacterial taxa between bats with different food types than between insectivorous *N. noctula* and *V. murinus* species (Figure 2 for genera, Figures S1–S4 for phylum, class, order, and family, respectively). There were 47 genera with an RA higher in fructivorous *C. perspicillata* and lower or even absent in insectivorous bats, while, on the other hand, there were 18 genera that were significantly more abundant in insectivorous bats compared to fructivorous species (Figure 2). Nonetheless, it is worth noting that there were also significant differences in the RA of some taxes between *N. noctula* and *V. murinus*. The most prominent differences were in *Kluyvera*, *Citrobacter*, *Mycoplasma*, *Hafnia-Obesumbacterium*, and *Leuconostoc*, which are significantly more abundant in *N. noctula* than in *V. murinus* and *C. perspicillata*, while *Enterobacter*, *Ureaplasma*, unclassified *Enterobacterales*, *Enterobacillus*, and *Weisella* were less abundant or absent in *N. noctula* gut microbiota (Figure 2).

### 2.2. Gut Microbiota Richness and Diversity

**Figure 3:**
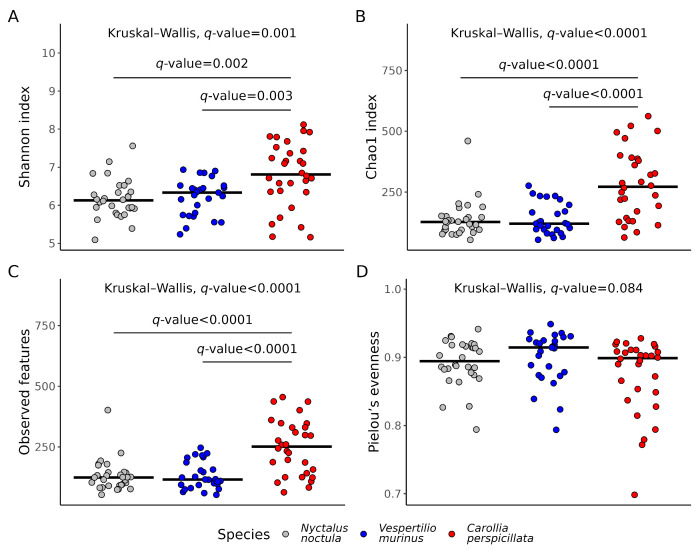
Alpha diversity metrics in samples from the *Nyctalus noctula*, *Vespertilio murinus*, and *Carollia perspicillata*. (**A**) Shannon index, (**B**) Chao1 index, (**C**) number of observed features, and (**D**) Pielou’s evenness. The results of the Kruskal–Wallis tests and multiple comparisons with the Mann–Whitney test were adjusted with the Benjamini–Hochberg procedure.

To compare microbial alpha diversity we used the Shannon, Chao1, and Pielou’s evenness indexes, as well as the total number of features in the acquired samples. According to the multiple comparison testing, there were statistically significant differences between the fructivorous *C. perspicillata* and the two insectivores *N. noctula* and *V. murinus* in all used alpha diversity metrics, except for Pielou’s evenness, while there were no statistical differences between the alpha diversity indexes in the gut microbiota between the two studied insectivore species (Figure 3).

**Figure 4:**
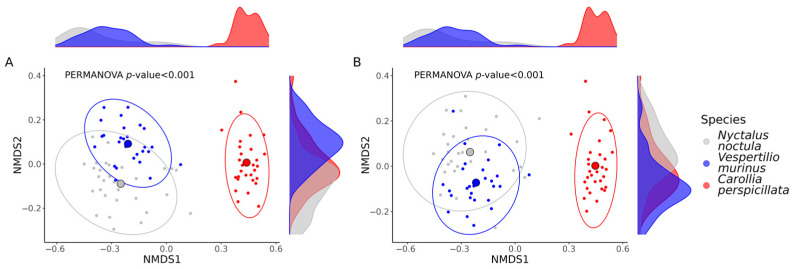
Non-metric multidimensional scaling (NMDS) ordination plot illustrating differences in beta diversity among samples from the *Nyctalus noctula*, *Vespertilio murinus*, and *Carollia perspicillata*. (**A**) NMDS plot showing a Bray–Curtis distance matrix. (**B**) NMDS plot showing a Jaccard distance matrix. *p*-values were calculated with the PERMANOVA test; the number of permutations was set to 1000.

To compare microbial beta diversity between studied bat species we used Bray–Curtis dissimilarity and Jaccard similarity indexes. As for the alpha diversity, the most significant differences were observed in the beta diversity between bats with different food types, while differences between the two insectivorous bats were less pronounced, as seen in the non-metric multidimensional scaling ordination plots (Figure 4).

### 2.3. Prediction of the Functional Pathways

**Figure 5:**
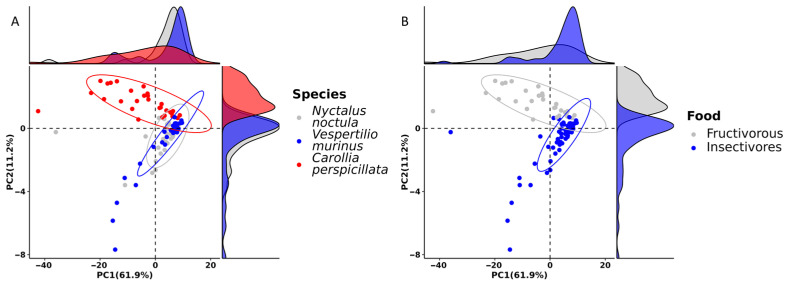
Principal component analysis (PCA) plot showing distances of predicted functional pathway abundances (**A**) between *Nyctalus noctula*, *Vespertilio murinus*, and *Carollia perspicillata* and (**B**) between bats with different food types.

To predict functional pathways based on the 16S rRNA metagenomic sequencing data, we used the PICRUSt2 pipeline [20], and the “ggpicrust2” R package [21] for the statistical analysis and the visualization of acquired results. First, we performed principal component analysis to investigate the overall differences in the distance of functional pathways. As a result, we observed that there were almost no differences in pathway distances between *N. noctula* and *V. murinus*, while the distances between *C. perspicillata* and insectivorous bats were more prominent (Figure 5).

**Figure 6:**
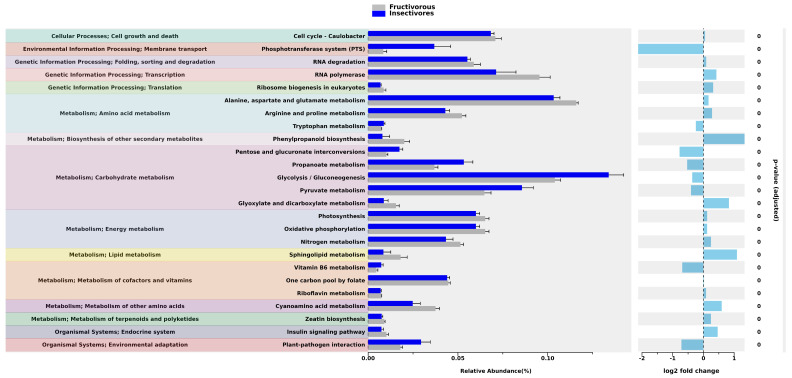
Results of the differential abundance analysis of predicted functional pathways in bats with different food types representing the top 25 pathways clustered into groups of functional categories. Error bar plots show a direct comparison of pathway abundance. Bar plots on the right represent log2 fold changes in the abundance of the pathways in bats with the different food types.

Based on the results of the differential abundance analysis of annotated functional pathways, there were 104 pathways for which differential abundance was closely tied with the food type. Most of the observed functional pathways were associated with microbial metabolisms, such as amino acids, carbohydrates, lipids, vitamins, and energy metabolism, although there were pathways associated with environmental interaction and genetic information processing. Figure 6 shows the top 25 functional pathways that have the most significant differential abundance between bats of different food types. Tables S1 and S2 contain full information about differentially abundant functional pathways in bats of different food niches and studied species.

## 3. Discussion

In this study, we investigated the gut microbiota composition and alpha/beta diversity of *N*. *noctula*, *V*. *murinus*, and *C*. *perspicillata*, predicted the microbiota functions, and made a statistical comparison of these features. Samples from *N*. *noctula* and *V*. *murinus* were obtained from bat rehabilitation centers in Rostov-on-Don and Moscow, respectively. The work of these centers is aimed at protecting the population of synanthropic bats, especially during fall and winter. Synanthropic insectivorous bats play a crucial role in the ecosystems of urban areas as they are natural controllers of agricultural pests [22,23]. In a recent study, Nsengimana et al. determined that 60% to 78% of the diet of insectivorous bats consists of agricultural pests [24]. Aguiar et al. calculated the value of pest suppression by synanthropic insectivorous bats and concluded that bats are saving USD 94 per hectare of cornfields and overall USD 390.6 million per harvest in Brazil [25]. These studies prove the beneficial role of bats in the human ecosystem; however, the process of urbanization may be detrimental to the bat population [26]. Due to their small size and ability to fly, bats are often found in households, as there are few places in cities where bats can safely roost and hibernate. When people find a bat or an entire colony, they tend to destroy them, putting the lives of these animals at risk. In some cases, bats are unable to survive hibernation, as the temperature conditions in some households could be extremely low. To save the population of synanthropic bats and the urban ecosystems, volunteers bring animals to bat rehabilitation centers, where the animals are fed insects, receive qualified veterinary care, and are placed in controlled hibernation at temperatures of 4–10 °C for the winter period before they are released in spring. In addition to the primary purpose of bat rehabilitation centers, they provide unique opportunities to study bats [27,28].

Samples from *C*. *perspicillata* were obtained from the Moscow Zoo. The natural habitat of this bat species is Central America, South America, and the Antilles Islands, and it is one of the most common bat species in zoos [29,30]. *C*. *perspicillata* bats were included in our study as the reference for the comparison with the gut microbiota of synanthropic bats, as the microbiota of this bat species was studied before [31,32]. Carrillo-Araujo et al. studied the gut microbiota in intestinal biopsies obtained from three *C*. *perspicillata* bats and found that the most abundant phyla in their gut microbiota were Pseudomonadota, Mycoplasmatota, Bacillota, and Cyanobacteria, which is generally consistent with our study, except for Mycoplasmatota, as we did not identify this phylum in the studied bats of the same species. Also, Bacteroidota were several times more abundant in our study. Interestingly, and entirely in line with our own observations, the authors found that Cyanobacteria were observed only in *C*. *perspicillata* among all other bat species [31]. The presence of Cyanobacteria in *C*. *perspicillata* is obviously linked to their food preferences; however, it is unknown why this phylum was observed only in this fructivorous bat species in previous studies. Lemieux-Labonté et al. studied the skin microbiota of *C*. *perspicillata*, which on the phylum level also corresponds to the gut microbiota of this species, as they observed Pseudomonadota, Bacillota, Bacteroidota, and Cyanobacteria [32], although their RA could differ among different body sites. Nevertheless, there should be a connection between gut and skin microbiota in bats, as feces could sometimes stick to the bats’ skin and affect skin microbiota composition, especially in the captive environment.

While there are some data on the microbiota of *C*. *perspicillata*, the data for *N*. *noctula* and *V*. *murinus* are very limited. The only study we found was conducted by Corduneanu et al., who reported the heart microbiome of insectivorous bats from Central and Southeastern Europe, including *N*. *noctula* [33]. In our study, we found that the RAs of at least 10 bacterial genera in the gut microbiota of *N*. *noctula* and *V*. *murinus* are significantly different between the two insectivores, while there were a minimum of 60 genera for which the RAs were different in *C*. *perspicillata* compared to the studied insectivorous bats. These results reveal much higher similarities in gut microbiota composition between synanthropic *N*. *noctula* and *V*. *murinus* from Rostov-on-Don and Moscow than between zoo *C*. *perspicillata* and these two bat species, although *V*. *murinus* was also collected in Moscow. This was confirmed by the results of alpha and beta diversity analyses, in which the differences between fructivorous and insectivorous bats appeared more prominent and significant than between the two insectivores. In our study, the food type appeared to be much more closely associated with the differences in the gut microbiota of studied bats than their species, supporting the hypothesis that the gut microbiota in bats is not only host phylogeny-specific [31]. Phillips et al. also observed that the gut microbiota community in fructivorous bats from Guatemala and Arizona was characterized as significantly more diverse than in insectivorous bats [14], which fully correlated with our results. This dependence of gut microbiota alpha and beta diversity on the food type is typical of mammals. Ley et al. demonstrated that the gut microbial diversity of herbivore mammals is significantly greater than that of carnivores [34]. However, it is worth mentioning that due to the anatomical and physiological features of the bat GIT, as well as the dependence of its microbiota composition on the environment, the relationship between food type and gut microbial diversity could be more prominent in bats.

Another important feature includes a markedly lower RA of facultative or obligatory anaerobic bacteria in the gut microbiota of *N*. *noctula* and *V*. *murinus* compared to *C*. *perspicillata*, as shown by the differential abundance analysis on the phylum and class levels. For example, the higher RA of Bacteroidota phylum and *Bacteroides* genus was significantly more associated with the gut microbiota of *C*. *perspicillata* compared to insectivorous *N*. *noctula* and *V*. *murinus*. Bacteroidota is a key taxon for human gut microbiota, and this phylum makes up the majority of the bacterial taxa present in the human intestinal microbiota, which is provided by the favorable anaerobic conditions in the human colon [35]. In comparison, bats have lower anaerobic volumes in the intestines, resulting in less favorable conditions for the strict anaerobic microbiota community found in other animal species. We did not find detailed data on anaerobic volumes of GIT in *N*. *noctula* and *V*. *murinus*, although the food transition time and maximum retention time in the GIT of these bat species are relatively short compared to other mammals, which may also indicate less favorable conditions for gut anaerobic bacteria [36]. The unique properties of the microbiota may also stem from the ability to fly, leading to increased energy metabolism and distinctive immune responses, as well as the GIT features [11,37,38]. In this study, we also identified lactic acid bacteria in bats such as *Pedicoccus*, *Latilactobacillus*, and *Lactococcus*, which were more abundant in *N*. *noctula* and *V*. *murinus* and less common or even absent in *C*. *perspicillata*. Lactic acid bacteria play a significant role in animal welfare, as they have immunomodulatory properties [39]. However, the immune system of bats significantly differs from other animals, which allows them to co-exist with dangerous pathogens due to limited inflammatory response and also provides them with tolerance to malignant tumors [40,41]. Some bat species, such as *N*. *noctula* and *V*. *murinus*, hibernate during winter, which also contributes to their immunity-associated features, such as longevity [42,43]. The role of lactic acid bacteria in all of these features is not yet investigated, but we speculate that revealing new mechanisms of interaction between lactic acid bacteria and the bat immune system could provide new insights into understanding complex relationships between bacteria, host organisms, and even viruses [44].

Notably, in synanthropic bats, we identified bacterial genera that could have an important role in public health and animal welfare, such as *Bacteroides*, *Enterobacter*, *Clostridiaceae*, *Enterococcus*, *Ureaplasma*, *Faecalibacterium*, *Helicobacter*, *Mycoplasma*, *Esherichia-Shigella*, and *Proteus*. Some of these bacteria are considered to be potentially pathogenic to humans and animals [45,46,47], although their pathogenic potential [47,48,49] can only be uncovered by additional studies. Most importantly, as these genera are common for farm and domestic animals, and also for humans, they could be involved in spreading antibiotic resistance genes in urban areas. For example, in recent studies of *Enterobacter* spp. isolated from bat fecal samples, the resistance of these bacteria to ampicillin and amoxicillin was reported in bats from Brazil [50], and the resistance to amoxicillin, ampicillin, amoxicillin-clavulanic acid, aztreonam, cefotaxime, cefepime, ceftazidime, and many other antibiotics was reported in bats from Gabon [51]. In another example, *Enterococcus* spp. isolated from rectal samples of bats in Spain showed resistance to erythromycin, quinupristin-dalfopristin, and ciprofloxacin [52], while Nowakiewicz et al. reported that *Enterococcus faecalis* isolated from guano samples and rectal swabs from bats in Poland displayed resistance to chloramphenicol, ciprofloxacin, erythromycin, gentamycin, kanamycin, rifampin, streptomycin, and tetracycline [53]. Moreover, the presence of some common antibiotic-resistance genes, such as blaCTX-M15, in bats from different populations in Nigeria [54], Gabon [51], Poland [53], and Peru [55] marks the global role of bats in spreading antibiotic-resistant genes. However, a scoping review identified *E. coli* as the most significant taxon involved in bat-derived antimicrobial resistance spread. However, in our study, the *Escherichia* genus was among the least abundant taxa in all three studied bat species. Nonetheless, this does not necessarily imply that the studied bats are unable to contribute to the spread of antibiotic resistance. This aspect requires further investigation.

According to the principal component analysis, the diversity of predicted functional pathways in the gut microbiota of *N*. *noctula* and *V*. *murinus* showed more similarities compared to *C*. *perspicillata*. As expected, these results demonstrate that gut microbiota features, including microbial metabolism, strongly depend on bat diet [56,57]. The RA of predicted functional pathways in the gut microbiota of studied bats is in agreement with previous studies. Ingala et al. conducted a comparison of microbial functional pathways based on the 16S rRNA sequenced gut microbiota data from 545 bats, which were obtained from multiple studies. In those studies, 360 and 141 of the samples were from insectivorous and fructivorous bats, respectively, while the remaining 44 samples were from bats of different food niches (sanguivorous, omnivorous, and carnivorous) [58]. The researchers found that the RA of at least 29 predicted functional pathways were enriched and significantly different between bats with these two food niches. Interestingly, in fructivorous bats, pathways were observed that could be associated with the production of essential amino acids, such as methionine, valine, isoleucine, and tryptophan. The authors suggested that the gut microbiota of fructivorous bats could compensate for the lack of essential amino acids for the host that could not be obtained through the food, which marks host-microbiota relationships in bats [58]. In our study, predicted pathways for amino acids in the gut microbiota of *C*. *perspicillata* were also significantly enriched in this bat species compared to insectivorous bats, although they were not essential (alanine, aspartate, glutamate, arginine, proline) [59]. The only differentially abundant essential amino acid synthesis pathway that could be possibly due to the gut microbiota was tryptophan synthesis, which was more abundant in insectivorous bats. The other major groups of functional pathways, for which the RA significantly differed among bats with different diet types in our study, were associated with carbohydrate and energy metabolism. The most prominent differences were in glycolysis/gluconeogenesis, which is significantly enriched in insectivorous bats compared to fructivorous. Given that the microbiota could be involved in the host metabolism, it is obvious that the gut microbiota of insectivorous bats could provide the host with the necessary carbohydrates during the microbial metabolism, which are already delivered in high concentrations in fruits consumed by fructivorous bats [60,61]. We should also note that the gut microbiota of the studied bats could be involved in the synthesis of some essential vitamins, such as vitamin B5 and riboflavin. Functional microbial pathways associated with vitamin synthesis were also previously observed in the gut microbiota of bats [58]. One of the interesting features that we noted in the predicted pathways was the potential impact of gut microbiota on genetic information processing, particularly, on transcription, which may be related to the pro-mutagenic properties of the lactic acid gut microbiota of bats that we observed previously [44]. However, this hypothesis requires further research.

We performed a differential abundance analysis of microbiota composition and functional pathway data with the MaAsLin2 package [19] after filtering the least abundant taxa/pathways. According to the results of the bioinformatic benchmarking study of Nearing et al., MaAsLin2 showed the most consistent results in avoiding false positives, but only with filtered data [62]. Some other studies of the bat gut microbiota relied on other methods of differential abundance analysis, so this should be taken into account when comparing the results of the published studies. Although it is clear that the gut microbiota of bats is very diverse and varies with the environment, the differences are evident with different analysis methods [18,63,64].

The sampling from synanthropic bats was conducted after hibernation in bat rehabilitation centers. Thus, we speculate that we observed bacteria that survived low temperatures within the bats’ bodies. Our recent study shows that hibernation results in the decrease of the gut microbiota diversity in bats, as food deprivation and low body temperature create unfavorable conditions for gut microbes [65]. Therefore, it is important to mention that the diversity of gut microbiota of studied insectivorous bats could have been decreased, as they awoke from hibernation at the moment of the study. Also, we should mention that the gut microbiota composition of the studied fructivorous and insectivorous bats was very diverse, although all of these bats were captive or semi-captive when sampled. This could mean that the gut microbiota of the studied bats was not grossly altered by humans, as all three bat species were in close contact with volunteers, veterinarians, biologists, and rehabilitation specialists, especially for the *C*. *perspicillata* colony in the zoo. However, further studies may reveal how close human contact impacts the gut microbiota of bats, which would require sampling from bats on their arrival [27,66].

## 4. Materials and Methods

### 4.1. Sampling

Sampling was conducted in February–March 2022 at the Bat Rehabilitation Center of Don State Technical University (Rostov-on-Don, Russia), the Bat Rehabilitation Center of the Moscow Zoo (Moscow, Russia), and the Experimental Department of Small Mammals of the Moscow Zoo (Moscow, Russia). In the study, 3 bat species were included, with 89 bats in total (Table 1).

All of the studied insectivorous bats were in an active state and recently arrived at the bat rehabilitation center facilities. Sampling of fecal samples from insectivorous species was performed before they were put into hibernation. A fructivorous bat colony was kept collectively at the zoo facility and consisted of captive-born individuals. A minimum of 0.5 g of fecal samples were collected from each bat and placed in sterile tubes with DNA/RNA Shield (Zymo Research, Irvine, CA, USA) and transported to the laboratory at room temperature.

### 4.2. Gut Microbiota Composition and Metagenomes Functions Prediction

Amplicons of the V3–V4 region of the 16S rRNA gene were sequenced to determine microbiota composition. In short, from the fecal samples, DNA was isolated with QIAamp Fast DNA Stool Mini Kit (Qiagen, Hilden, Germany), amplified with barcoding, pooled, and subsequently sequenced with the Illumina MiSeq sequencing system according to the manufacturer’s instructions (Illumina, San Diego, CA, USA). The Binary Base Call text-based format for storing the biological sequence and corresponding quality scores pipeline (BCL2FASTQ, version 1.8.3, Illumina, San Diego, CA, USA) was used to convert the sequences into a text-based format for storing biological sequence and corresponding quality score (FASTQ) files after quality checking. The results were then analyzed using the Quantitative Insights Into Microbial Ecology 2 (QIIME-2, version 2023.5) software [67]. For the data analysis, we performed denoising with DADA2 [68] and used the Silva database (version 138 (available online: https://www.arb-silva.de/documentation/release-138/, accessed on 11 July 2023)) as a reference 16S rRNA database for the classification of amplicon sequence variants [69,70]. A prediction of metagenome functions was conducted using the PICRUSt2 (version 2.5.2.) [20]. Annotations of functional pathways were performed with the KEGG database [71].

### 4.3. Statistical Analysis

The programming language R v4.2.3 (R Foundation for Statistical Computing, Vienna, Austria) was used for statistical analyses. MaAsLin2 package was used for the differential abundance analysis [19]. The *C. perspicillata* species was used as a reference factor for the linear model analysis. Before differential abundance analysis, we performed filtering by applying a minimal 10% threshold for the prevalence of the taxa to obtain more robust results [62]. Based on the acquired counts of data, the RA of taxa was calculated. Taxa with an RA higher than 1% were used for the differential abundance analysis. The rarefaction threshold for the alpha and beta diversity analyses was 2500 sequences/sample. The Kruskal–Wallis test was performed to determine differences in the alpha diversity matrix. PERMANOVA with the adonis function from the “vegan” package was used to determine differences in beta diversity distances (the number of permutations was set to 1000). We used the “ggplot2” package for the visualization of the differential abundance analysis results as volcano plots and heatmaps, alpha diversity analysis results as jitter plots, and beta diversity analysis as non-metric multidimensional scaling ordination plots [72]. The PICRUSt2 outcome was analyzed and visualized with the”ggpicrust2” package [21]. Differential abundance analysis of predicted functional pathways was carried out in the same way as with the taxa RA described above. Multiple comparisons were performed with the Mann–Whitney test, and the results were adjusted with the Benjamini–Hochberg false discovery rate. Adjusted *p*-values (*q*-values) were considered significant at *q* < 0.05.

## 5. Conclusions

In conclusion, in this study, we provide the first data on gut microbiota composition and potential functional activity of two synanthropic species, *N. noctula* and *V. murinus*, from Rostov-on-Don and Moscow, respectively, and performed a direct comparison of these features with *C. perspicillata* bats from a zoo colony. The observed features point to possible host-microbiota interactions and the significance of studied bat gut microbiota for public health and animal welfare. The overall results are consistent with the previous studies of bat gut microbiota, although all bat species studied here showed some unique microbiota features compared to previous reports, which is reasonable given their different geographical location and climatic environments.

## Figures and Tables

**Table 1:** Bat species, sampling location site, and number of animals included in the study.

Bat Species	Food Type	Sampling Location Site	Number
*Nyctalus noctula*	Insectivorous	Bat Rehabilitation Center of Don State Technical University, Rostov-on-Don	30
*Vespertilio murinus*	Insectivorous	Bat Rehabilitation Center of Moscow Zoo, Moscow	29
*Carollia perspicillata*	Fructivorous	Experimental Department of Small Mammals of Moscow Zoo, Moscow	30

## Data Availability

All sequences associated with this study have been deposited int the National Center for Biotechnological Information’s Short Read Archive and are available under BioProject ID PRJNA1012124.

## Supplementary Materials

The following supporting information can be downloaded at: https://www.mdpi.com/article/10.3390/ijms242417301/s1.
