# Supplementary material for: Gut Microbiota Composition of Insectivorous Synanthropic and Fructivorous Zoo Bats: A Direct Metagenomic Comparison
Source: Int J Mol Sci. 2023 Dec 9;24(24):17301. doi: 10.3390/ijms242417301 (PMC10744024; doi:10.3390/ijms242417301)
Supplement: Supplementary file 1 [file ijms-24-17301-s001.zip › IJMS_supplementary_figures.pdf]

Supplementary Figures

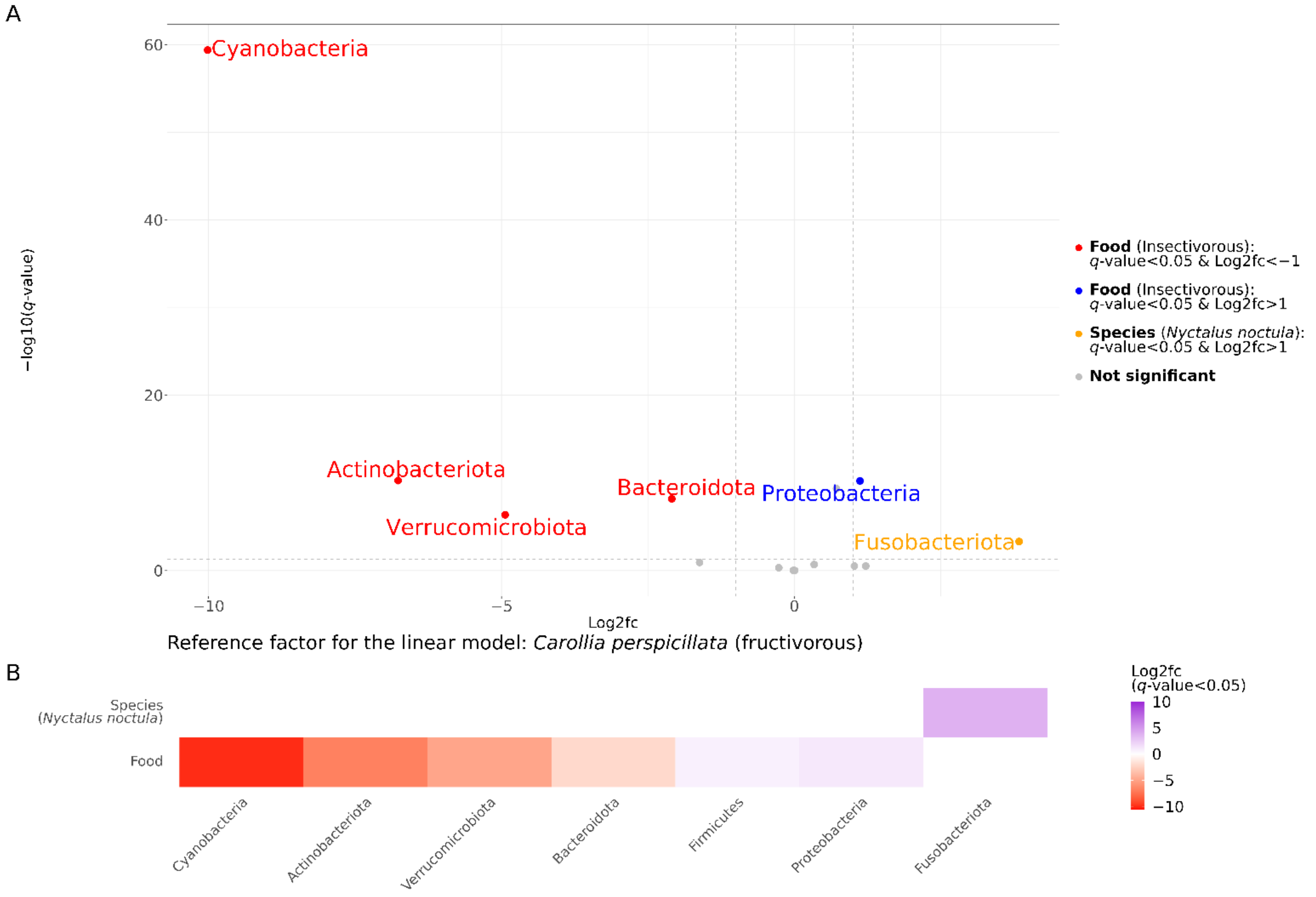

Figure S1. Results of the differential abundance analysis of bacterial phyla identified in fecal samples of insectivorous (*Nyctalus noctula* and *Vespertilio murinus*) and fructivorous (*Carollia perspicillata*) bats. The fructivorous species *Carollia perspicillata* was used as a reference factor for the linear model analysis. (A) Volcano plots with phyla, the relative abundance (RA) of which had significant associations with food type and studied bat species. Blue dots represent phyla for which RA was strongly associated with the insectivorous diet of bats. Red dots represent phyla that were significantly less abundant or even absent in insectivorous bats compared to fructivorous ones. Yellow dots show phyla that had a greater RA in *Nyctalus noctula* compared to *Carollia perspicillata* and *Vespertilio murinus*, while green dots represent phyla, which were less abundant in *Nyctalus noctula* compared to *Carollia perspicillata* and *Vespertilio murinus*. (B) Heatmap with phyla, the RA of which had significant associations with food type and studied bat species. In the top row, purple cells in the heatmap represent phyla for which RA was significantly higher in *Nyctalus noctula* compared to *Carollia perspicillata* and *Vespertilio murinus*, while orange cells show the opposite. In the second line, purple cells represent phyla with RA significantly higher in the insectivorous bats studied compared to the fructivorous bats, and orange cells show phyla, which are significantly less abundant or absent in insectivorous bats compared to fructivorous bats.

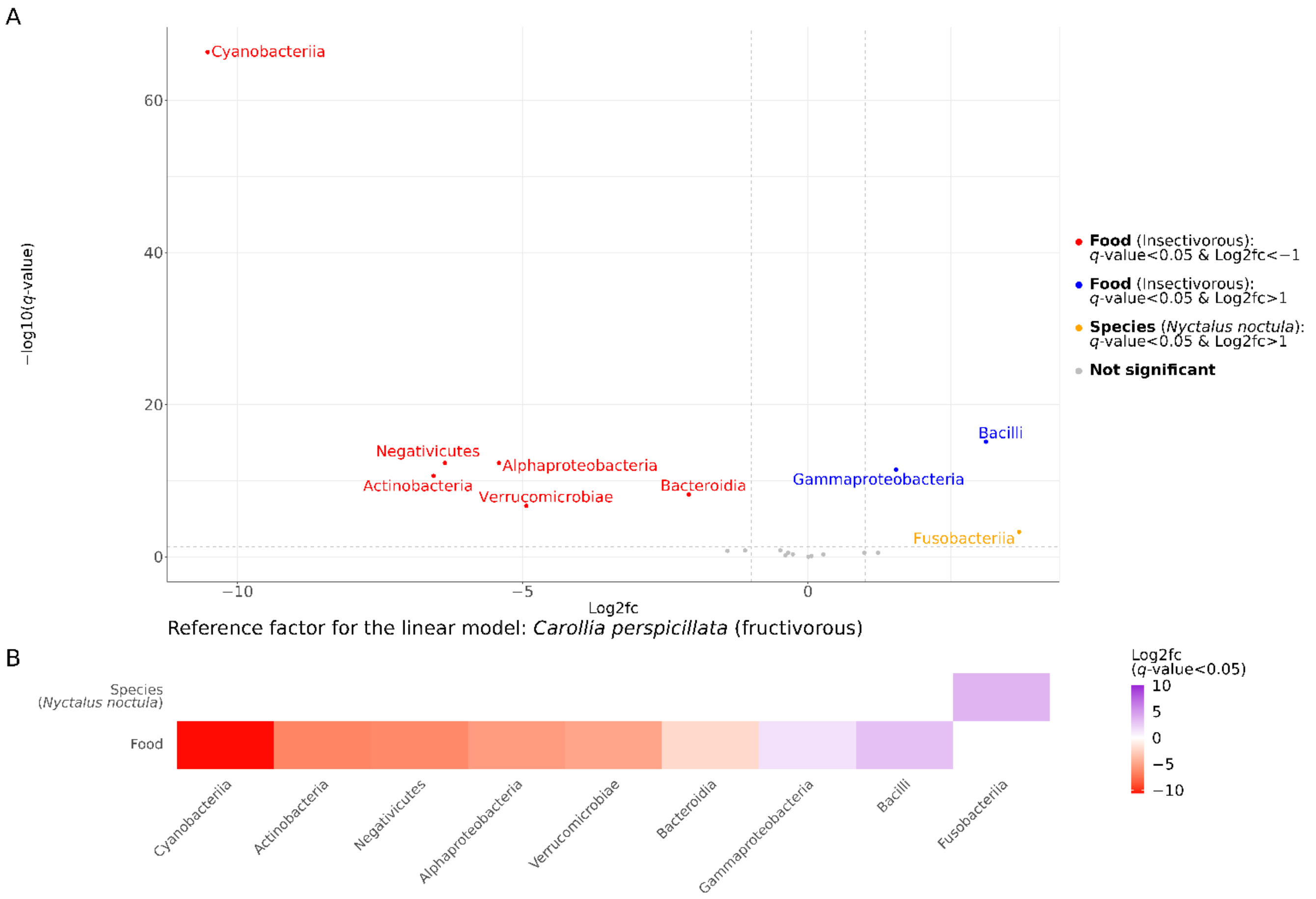

Figure S2. Results of the differential abundance analysis of bacterial classes identified in fecal samples of insectivorous (*Nyctalus noctula* and *Vespertilio murinus*) and fructivorous (*Carollia perspicillata*) bats. The fructivorous species *Carollia perspicillata* was used as a reference factor for the linear model analysis. (A) Volcano plots with classes, the relative abundance (RA) of which had significant associations with food type and studied bat species. Blue dots represent classes for which RA was strongly associated with the insectivorous diet of bats. Red dots represent classes that were significantly less abundant or even absent in insectivorous bats compared to fructivorous ones. Yellow dots show classes that had a greater RA in *Nyctalus noctula* compared to *Carollia perspicillata* and *Vespertilio murinus*, while green dots represent classes, which were less abundant in *Nyctalus noctula* compared to *Carollia perspicillata* and *Vespertilio murinus*. (B) Heatmap with classes, the RA of which had significant associations with food type and studied bat species. In the top row, purple cells in the heatmap represent classes for which RA was significantly higher in *Nyctalus noctula* compared to *Carollia perspicillata* and *Vespertilio murinus*, while orange cells show the opposite. In the second line, purple cells represent classes with RA significantly higher in the insectivorous bats studied compared to the fructivorous bats, and orange cells show classes, which are significantly less abundant or absent in insectivorous bats compared to fructivorous bats.

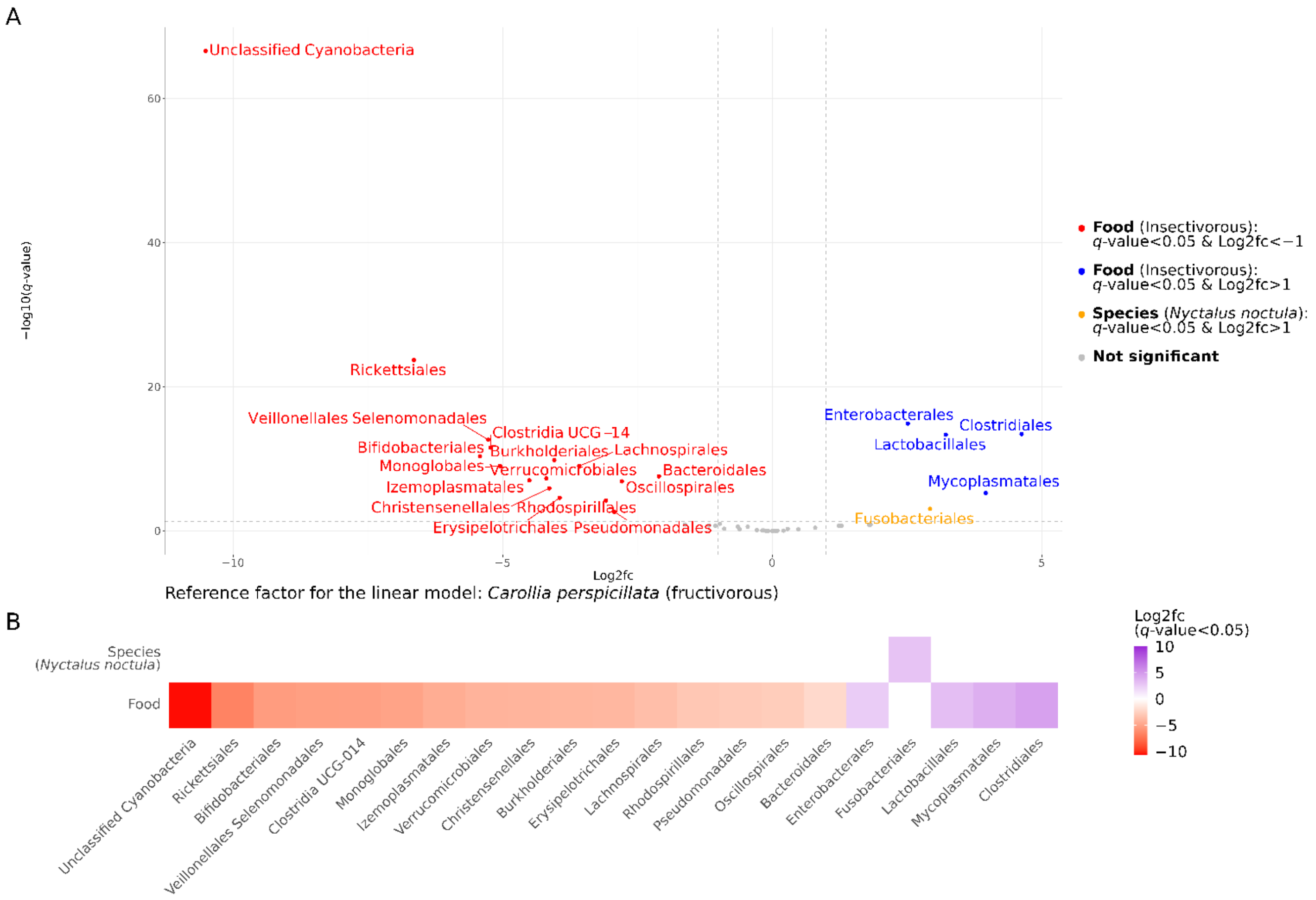

Figure S3. Results of the differential abundance analysis of bacterial orders identified in fecal samples of insectivorous (*Nyctalus noctula* and *Vespertilio murinus*) and fructivorous (*Carollia perspicillata*) bats. The fructivorous species *Carollia perspicillata* was used as a reference factor for the linear model analysis. (A) Volcano plots with orders, the relative abundance (RA) of which had significant associations with food type and studied bat species. Blue dots represent orders for which RA was strongly associated with the insectivorous diet of bats. Red dots represent orders that were significantly less abundant or even absent in insectivorous bats compared to fructivorous ones. Yellow dots show orders that had a greater RA in *Nyctalus noctula* compared to *Carollia perspicillata* and *Vespertilio murinus*, while green dots represent orders, which were less abundant in *Nyctalus noctula* compared to *Carollia perspicillata* and *Vespertilio murinus*. (B) Heatmap with orders, the RA of which had significant associations with food type and studied bat species. In the top row, purple cells in the heatmap represent orders for which RA was significantly higher in *Nyctalus noctula* compared to *Carollia perspicillata* and *Vespertilio murinus*, while orange cells show the opposite. In the second line, purple cells represent orders with RA significantly higher in the insectivorous bats studied compared to the fructivorous bats, and orange cells show orders, which are significantly less abundant or absent in insectivorous bats compared to fructivorous bats.

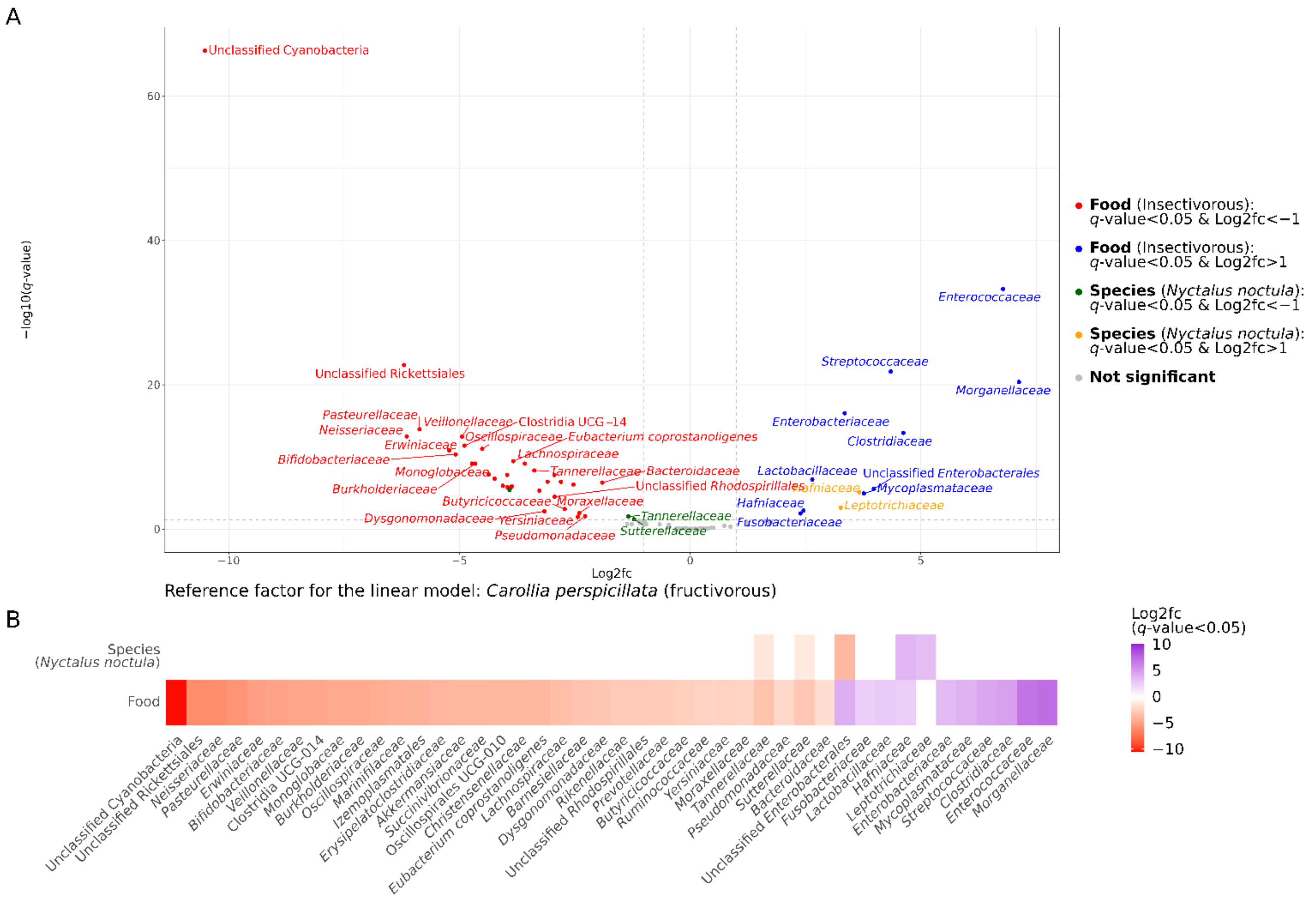

Figure S4. Results of the differential abundance analysis of bacterial families identified in fecal samples of insectivorous (*Nyctalus noctula* and *Vespertilio murinus*) and fructivorous (*Carollia perspicillata*) bats. The fructivorous species *Carollia perspicillata* was used as a reference factor for the linear model analysis. (A) Volcano plots with families, the relative abundance (RA) of which had significant associations with food type and studied bat species. Blue dots represent families for which RA was strongly associated with the insectivorous diet of bats. Red dots represent families that were significantly less abundant or even absent in insectivorous bats compared to fructivorous ones. Yellow dots show families that had a greater RA in *Nyctalus noctula* compared to *Carollia perspicillata* and *Vespertilio murinus*, while green dots represent families, which were less abundant in *Nyctalus noctula* compared to *Carollia perspicillata* and *Vespertilio murinus*. (B) Heatmap with families, the RA of which had significant associations with food type and studied bat species. In the top row, purple cells in the heatmap represent families for which RA was significantly higher in *Nyctalus noctula* compared to *Carollia perspicillata* and *Vespertilio murinus*, while orange cells show the opposite. In the second line, purple cells represent families with RA significantly higher in the insectivorous bats studied compared to the fructivorous bats, and orange cells show families, which are significantly less abundant or absent in insectivorous bats compared to fructivorous bats.

Table S1. Results of the differential abundance analysis of predicted functional pathways in bats with different food types. The table contains names and descriptions of predicted functional pathways in which enrichment was significantly different between bats with different food niches.

Table S2. Results of the differential abundance analysis of predicted functional pathways in *Nyctalus noctula*, *Vespertilio murinus*, and *Carollia perspicillata*. The table contains names and

descriptions of predicted functional pathways in which enrichment was significantly different between *Nyctalus octule*, *Vespertilio murinus,* and *Carollia perspicillata*.
